# Vitreous opacities after intravitreal triamcinolone injection- a case report

**DOI:** 10.1186/s12886-018-0818-y

**Published:** 2018-06-20

**Authors:** Dhanashree Ratra, Vineet Ratra, Haard Shah

**Affiliations:** 1 Department of Vitreoretinal Diseases, Medical Research Foundation, Sankara Nethralaya, 41, College Road, Chennai, 600006 India; 2 Department of Comprehensive Ophthalmology, Medical Research Foundation, Sankara Nethralaya, 41, College Road, Chennai, 600006 India

**Keywords:** Intravitreal injections, Triamcinolone, Preservative, Vitreous opacities, Macular edema

## Abstract

**Background:**

We report occurrence of peculiar tiny white thread like vitreous opacities after intravitreal triamcinolone injection. These persisted without any change for over a year. We ascribe them to aggregation of triamcinolone crystals due to the purification methods.

**Case presentation:**

Seven patients (8 eyes) with macular edema developed tiny whitish thread like opacities in the vitreous 2–3 months after undergoing an intravitreal injection of triamcinolone acetonide preparation containing benzyl alcohol as preservative. These opacities persisted unchanged for more than a year. The follow up ranged from 91 to 425 days. Vitreous tap was done in one patient which was negative for infection. All patients initially showed improvement but needed re-treatment for recurrence. One patient developed steroid induced rise in intraocular pressure. Microscopic examination of the drug revealed large string like aggregates of triamcinolone crystals.

**Conclusions:**

We hypothesize the possibility of aggregation of triamcinolone crystals into string like structures probably due to the purification methods used during manufacture which led to these thread like opacities in the vitreous.

## Background

Intravitreous triamcinolone acetonide (IVTA) has found widespread application for a variety of posterior segment pathologies which cause macular edema. Its therapeutic efficacy has been well demonstrated in clinical trials [1–3]. And its cost effectiveness has made IVTA a widely used agent. Several drug-related and injection-related complications have been reported ranging from localized subconjunctival hemorrhage at the site of injection to serious sight threatening problems [4]. Triamcinolone acetonide is a long acting steroid which is available as a suspension of crystals in benzyl alcohol. The particulate nature of the drug and the lack of preservative free formulation can give rise to deposits on the retina and in the vitreous cavity. The triamcinolone crystals appear as shiny white deposits in the vitreous and have been reported to last for more than 120 days after injection [5]. We report the occurrence of peculiar small thread like opacities in the vitreous which persisted for over a year after the IVTA injection.

## Case presentation

We examined seven patients (8 eyes, one bilateral) out of total 15 patients who had received IVTA injection from September 2015 to September 2016. The study adhered to the tenets of the Helsinki Declaration and was approved by the institutional review board of Medical Research Foundation, Chennai. Written informed consent was obtained from every patient. All patients underwent pre-injection detailed eye examination including best corrected visual acuity, intraocular pressure evaluation and fundus evaluation including macular optical coherence tomography. Triamcinolone acetonide, 0.05 ml (2 mg), (Kenacort, Abbott healthcare private limited, Mumbai, India containing triamcinolone acetonide 40 mg and benzyl alcohol 0.9% *w*/*v* as preservative in aqueous base) was withdrawn from the vial and injected directly intravitreally through the pars plana using a 26 gauge needle. The preservative free triamcinolone was not available to us. One eye received 4 mg IVTA. The injections were performed in the operation theatre with complete aseptic precautions. Before the injection a drop of topical lignocaine and a drop of 5% betadine was instilled in the eye. After injection betadine eye drops were applied and eye was patched for half an hour. No pre or post injection antibiotics were used. All the patients were examined the day after and again 3 days later to rule out increased intraocular pressure or any suspected intraocular infection. Thereafter all the patients were examined at monthly interval or depending upon the therapeutic response. As per the routine protocol, all the patients underwent vision check, refraction, slit lamp examination, IOP measurement by applanation tonometer, pupillary dilation followed by retinal evaluation with indirect ophthalmoscopy and an optical coherence tomography scanning.

**Fig. 1:**
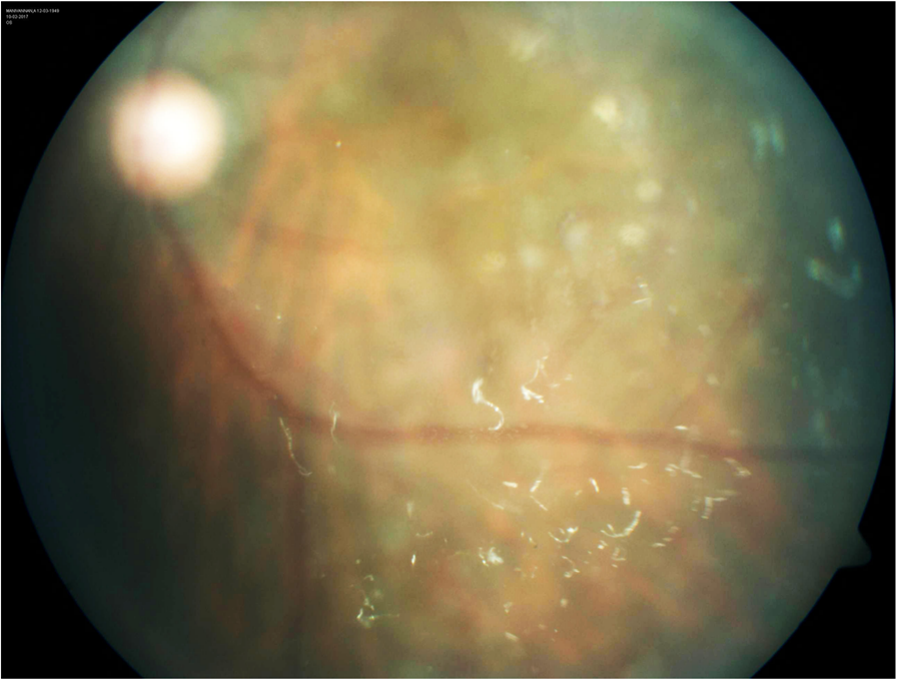
Tiny white thread like opacities in the vitreous following injection of triamcinolone acetonide

**Fig. 2:**
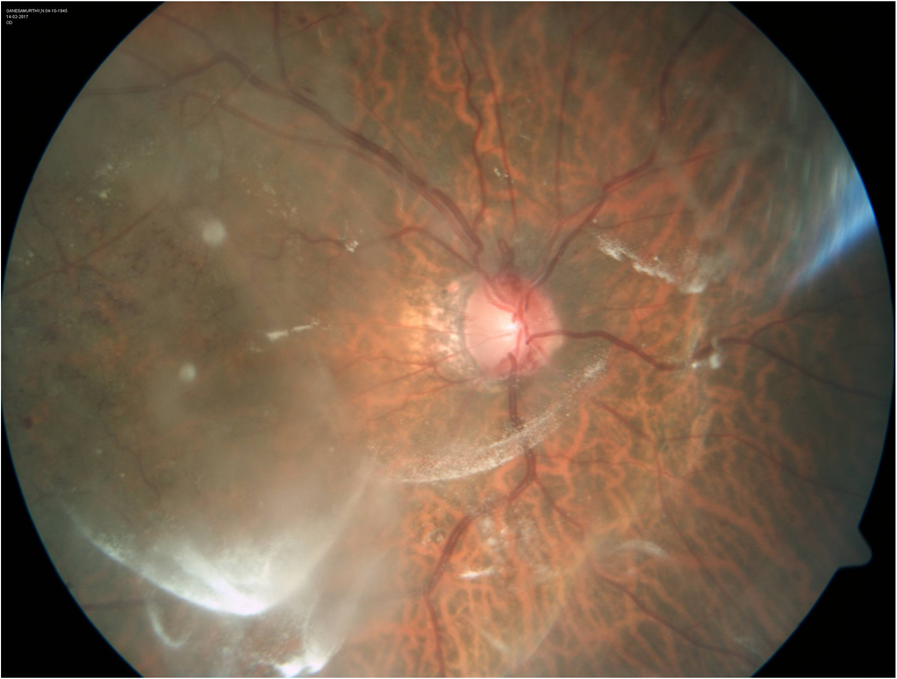
Round particulate opacities of freshly injected triamcinolone one day after the injection

On indirect ophthalmoscopy, we noticed peculiar tiny, thread like, whitish, mobile opacities in the vitreous, 2–3 months after the IVTA injection in 8 eyes of 7 patients (Fig. 1) which were different from the powdery, rounded, particulate opacities of freshly injected triamcinolone (Fig. 2). Table 1 lists the details of the patients. Except one, all patients had received multiple prior anti-vascular endothelial growth factor or steroid injections. These opacities were noticed after a single injection of IVTA. Except for 1 patient with vitreous floaters, none had any prior notable vitreous changes. The IVTA injections were given at different time points and did not belong to the same batch of manufacture. There were no common factors linking these patients. Except one patient who developed steroid induced glaucoma, none others had any complications of the IVTA. There was good therapeutic response in all patients with immediate reduction of the macular edema. However, all required retreatment for recurrence of macular edema. They received either IVTA, dexamethasone implant or bevacizumab for further treatment. These thread-like opacities were seen to persist with hardly any change in all patients till the last follow up. The follow up ranged from 91 days till 425 days. The opacities did not resemble infectious endophthalmitis. Also the patients were comfortable with no signs of pain, redness or visual disturbance. We performed vitreous tap for 1 patient, which was negative for any microorganisms or cellular material. The bacterial and fungal cultures were negative. One of the injection vials were also subjected to bacterial and fungal cultures which were negative.

**Table 1:** Demographic details, indications and the number of days the vitreous opacities were seen to be persisting after the triamcinolone acetonide injection in the vitreous

Sr No	Age	Sex	Eye	Indication	Lens status	Procedure	Dose	Follow up of vitreous opacities in days	Other complications
1	66	M	OS	DME	Pseudophake	PE + IOL + IVTA	2 mg	91	None
2	59	M	OS	DME	Phakic	IVTA	4 mg	218	Steroid induced glaucoma
3	59	M	OD	DME	Phakic	IVTA	2 mg	188	Steroid induced glaucoma
4	43	F	OS	Pseudophakic-CME	Pseudophake	IVTA	2 mg	305	None
5	54	F	OS	Pseudophakic-CME	Pseudophake	IVTA	2 mg	189	None
6	66	M	OS	DME	Pseudophake	IVTA	2 mg	425	None
7	47	F	OS	STBRVO-CME	Pseudophake	IVTA	2 mg	216	None
8	68	M	OS	DME	Pseudophake	IVTA	2 mg	344	None

*OD* right eye, *OS* left eye, *DME* diabetic macular edema, *CME* cystoid macular edema, *STBRVO* superotemporal branch vein occlusion, *PE* phacoemulsification, *IOL* intraocular lens, *IVTA* intravitreal triamcinolone acetonide

## Discussion and conclusions

Various types of vitreous opacities can occur after IVTA injection. The triamcinolone acetonide, a low water soluble suspension, itself remains in the vitreous as white crystalline deposits. These can cause visual disturbances. On ultrasonography, they appear as clump like, point like, pseudo-membranous or as fine opacities in the subhyaloid space [6]. Triamcinolone is broken down into soluble metabolites 6 beta­hydroxy triamcinolone acetonide, 21­carboxy triamcinolone acetonide, 21carboxy ­6beta ­hydroxy triamcinolone acetonide. These generally clear spontaneously in about 30–60 days.

Both infectious and non-infectious types of endophthalmitis have been reported to occur following IVTA [7]. Bacterial endophthalmitis can occur with vitreous exudates, conjunctival injection, hypopyon and pain. This requires prompt treatment with intravitreal antibiotics. Often times the crystals of triamcinolone can migrate to the anterior chamber giving an appearance of a hypopyon [8].

An endophthalmitis-like reaction also has been reported following IVTA, wherein dense vitreous haze was seen occluding the vision but without the signs of classic infectious endophthalmitis [9]. This haze was seen to clear spontaneously over a few days. The reason for this was not clear but was thought to be due to spraying of the steroid inside the vitreous cavity while forcible injection through the small gauge needle which might have been jammed.

The preservative or the vehicle used for the triamcinolone preparation may also cause a toxic reaction. In animal studies, severe reaction leading to retinal necrosis has been seen due to the preservatives in various preparations of depot steroids [10]. In vitro studies have shown toxic effects of benzyl alcohol on human retinal pigment epithelial cells [11]. It is recommended to use the preservative free formulation or minimize the amount of preservative in the injection by keeping the injection standing for half an hour after it has been withdrawn in the syringe and then use only the sedimented crystals after discarding the supernatant fluid containing the preservative.

Silicone oil droplets have also been seen after an intravitreal injection. In the SCORE study [11], 33% of eyes treated with 1 mg of triamcinolone and 12% of eyes treated with 4 mg of triamcinolone had silicone oil droplets. However, this was due to the use of syringes with staked on needles. The problem disappeared following the change to syringes with luer lock needles.

Till now, there are no reports of such thread like tiny opacities as seen in our case series. A vitreous tap in one patient was negative and ruled out infection. Also, the fact that these patients were comfortable and in fact reported visual improvement with no change whatsoever in the number or appearance of these opacities, rule out the possibility of infection.

**Fig. 3:**
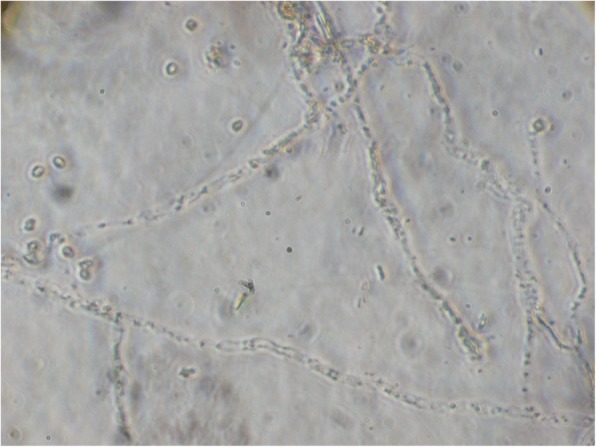
Photomicrograph of the drug showing thread like aggregates of the triamcinolone crystals

When we examined the drug under the microscope it revealed string like aggregates of the triamcinolone crystals (Fig. 3). The different methods used in the preparation of the triamcinolone can affect its crystal size, concentration and distribution. Kleinman et al. [12] conducted particle characterization analyses on different formulations of TA. They found variable crystals size and aggregation in different formulations. Szurman et al. [13] also noted that during the purification process, the crystal size and aggregation tended to differ with different filter parameters such as pore size and filter diameter. However, such string like aggregates have not been reported so far. The size of the aggregates determines whether the crystals will disperse in vitreous or gravitate down [14]. The number and size of the triamcinolone crystal aggregates have also been shown to be correlated with cytotoxicity, with large conglomerates being more cytotoxic [15]. For standardized uniformity, post-purification quality controls have been suggested.

In conclusion, seven patients are reported with development of tiny thread like vitreous opacities after an intravitreal injection of triamcinolone preparation with preservative. These opacities seem to be harmless, causing no visual disturbance or any other symptoms. However, they were seen to persist without any change for over a year.
